# Integrated IMRT vs segmented 3D-CRT of the chest wall and supraclavicular region for Breast Cancer after modified Radical Mastectomy: An 8-year follow-up

**DOI:** 10.7150/jca.51125

**Published:** 2021-01-10

**Authors:** Yutian Zhao, Jiahao Zhu, Xiaojun Zhang, Gang Wu, Yu Xu, Peipei Shen, Xianding Wei, Dong Kong, Shengjun Ji, Bo Yang

**Affiliations:** 1 Department of Radiotherapy and Oncology, Affiliated Hospital of Jiangnan University, Wuxi, Jiangsu 214000, P.R. China.; 2 Department of Radiotherapy and Oncology, Suzhou Municipal Hospital, Suzhou, Jiangsu 215000, P.R. China.

**Keywords:** breast cancer, modified radical mastectomy, intensity modulation, late toxicity

## Abstract

**Objective:** The purpose of this study was to evaluate the efficacy of two radiotherapy techniques for breast cancer patients with post-mastectomy. The intensity-modulated radiotherapy for treating the chest wall and regional nodes contoured as a whole planning target volume was compared with the conventional segmented 3-dimensional conformal radiotherapy undergoing modified radical mastectomy.

**Materials and methods:** Patients who received the two post-mastectomy radiation therapies were retrospectively analyzed. The chest wall and supra/infraclavicular region +/- internal mammary nodes were contoured as a whole planning target volume on the planning computed tomography. We evaluated differences in survival, recurrence, and late side effects between the integrated intensity-modulated radiotherapy group and the conventional segmented group.

**Results:** A total of 223 patients were recruited. The mean follow-up was 104.3 months. Of these patients, 129 received integrated radiotherapy and 94 patients received segmented radiotherapy. The 8-year disease-free survival rates were 86.0% and 73.4% for patients treated with integrated radiotherapy and traditional segmented radiotherapy, respectively (*P* = 0.022). The 8-year overall survival rates were 91.4% and 86.2% for patients treated with integrated radiotherapy and traditional segmented radiotherapy, respectively (*P* = 0.530). Multivariate analysis demonstrated that radiotherapy was an independent prognostic factor for disease-free survival. No significant difference was observed in late side-effects between the two groups.

**Conclusion:** Intensity-modulated radiotherapy for treating the chest wall and regional nodes contoured as a whole planning target volume reduces the recurrence rate for post-mastectomy breast cancer patients with tolerable toxicities.

## Introduction

Breast cancer is the most commonly diagnosed cancer and the leading cause of cancer death in women worldwide 1. Several randomized clinical trials demonstrated that adding chest wall and regional lymph node irradiation after modified radical mastectomy is beneficial for the disease-free survival (DFS) and overall survival (OS) of breast cancer patients with positive axillary lymph nodes 2-4. Intensity-modulated radiotherapy (IMRT) has been widely used in breast cancer patients after modified radical mastectomy. Our previous study confirmed that a post-mastectomy linac IMRT, with the chest wall and regional nodes contoured as a whole planning target volume (PTV), was dosimetrically feasible and well-tolerated by most patients 5. However, the differences in long-term prognosis and late side effects in patients treated with integrated IMRT and the conventional segmented 3-dimensional conformal radiotherapy (3D-CRT) are still unknown. This study was conducted to evaluate the differences in survival rates, recurrence, and late side effects between the two radiotherapy regimens.

## Materials and methods

### Patients

We retrospectively analyzed data from 223 patients with clinical stage II-III female breast cancer who received modified radical mastectomy, had positive axillary lymph nodes, and were treated with postoperative IMRT in the Affiliated Hospital of Jiangnan University and Suzhou Municipal Hospital from October 2009 to December 2013. Patients with male gender, the history of previous thoracic radiotherapy, the diagnosis of synchronous contralateral breast cancer (contralateral breast cancer was also diagnosed at the same time or within three months) and the neoadjuvant chemotherapy were excluded in this cohort. The review of data for this investigation was approved by the institutional review board of the Affiliated Hospital of Jiangnan University and Suzhou Municipal Hospital.

The data collected in our study including age at diagnosis, primary surgery and postoperative pathology information (grading, hormonal receptor status, human epidermal growth factor receptor 2 (HER2) overexpression status), adjuvant chemotherapy regimen with or without hormonal treatment, radiotherapy plan with or without internal mammary node irradiation (IMNI), DFS, OS, and post-radiotherapy late side effects in the skin, lungs, or shoulder joints. Breast cancer stage was categorized based on the 2018 TNM classification system (AJCC 8).

### Treatments

#### Surgery and adjuvant chemotherapy

All the patients recruited in this study underwent modified radical mastectomy and received level I/II axillary lymph node dissection routinely, with a median of 15 dissected lymph nodes (range 6-24). All the patients received postoperative chemotherapy because they had a positive lymph node. Anthracycline-based regimen EC (epirubicin 90 mg/m^2^ and cyclophosphamide 500 mg/m^2^ on day 1, every 3 weeks for 4 cycles) followed by taxanes (docetaxel 80 mg/m^2^ d1, every 3 weeks for 4 cycles) or anthracycline-based regimen FEC (fluorouracil 500 mg/m^2^, epirubicin 90 mg/m^2^, and cyclophosphamide 500 mg/m^2^ on day 1, every 3 weeks for 3 cycles) followed by taxanes (docetaxel 80 mg/m^2^, every 3 weeks for 3 cycles), with or without trastuzumab regimens, were used in the postoperative systemic therapy. All patients with positive hormone receptor received adjuvant hormone therapy. Tamoxifen or aromatase inhibitors were given according to the menopausal status. All patients with HER2 overexpression were additionally treated with trastuzumab.

#### Radiation therapy

Postoperative radiotherapy was applied after the completion of adjuvant chemotherapy. The detailed border of the target volume for the chest wall (CTVc) and the supraclavicular region (CTVs) was defined as in our previous study 5. The PTV for the chest wall (PTVc) consists of CTVc plus a 5-mm expansion and the CTVs plus a uniform 5-mm margin allover except towards the posterior edge formed the PTV of the supraclavicular region (PTVs). The PTVc and the PTVs merged into an integrated PTV. Organs at risk included the lung, heart, spinal cord, and the contralateral breast. Radiotherapy of internal mammary nodes was considered when the primary tumor's location was in the central or medial part of the breast or the high risks existed, including triple-negative breast cancer, positive axillary lymph nodes >4 or tumor size ≥5 cm.

In the integrated IMRT group, 6-MV x-rays were delivered using Varian linear accelerator. Six intensity-modulated radiation fields were used for the design of the integrated IMRT plan including three for the PTVc, two for the PTVs, and one for the entire PTV. A prescription dose of 50 Gy in 25 fractions was delivered.

In the conventional segmented treatment group, two tangential fields were used for the PTVc. The prescription dose was 50 Gy/25 fractions. For the PTV of the upper supraclavicular region, a mixed radiation of 6-MV x-rays, 16 Gy, and 9-MeV electrons, 34 Gy, was delivered in the anterior-posterior direction and abutted the radiation field of the PTVc with the half-field technique.

### Patient follow-up and outcomes

All patients were followed up every 3 months for the first 2 years, every 6 months in the third and once a year afterwards. Patients were monitored by physical examination, ultrasonography, laboratory analysis and radiologic imaging to detect recurrence. Recurrence in the ipsilateral chest wall or breast, ipsilateral axillary, infraclavicular, or supraclavicular nodes, or internal mammary nodes was defined as locoregional recurrence (LRR). DFS was defined as the period between the date of surgery and the time point of disease recurrence, death, or the last visit. OS was defined as the interval from the surgery to death or the last visit. Toxicity was graded according to the Common Toxicity Criteria for Adverse Events (CTCAE; version 4.0).

### Statistical analysis

Chi-square test or the Fisher's exact test was used to compare the characteristics between the two groups. For continuous variables, Student's t-test and the Wilcoxon rank-sum test was used. Kaplan-Meier curves were drawn for DFS and OS and compared using the log-rank test. Univariate and multivariate analyses were performed based on the Cox proportional-hazards regression model to identify the prognostic factors. Two-sided P values < 0.05 were considered significant. Statistical analysis was performed using SPSS version 24.0.0 (SPSS, Chicago, IL).

## Results

### Patient and treatment characteristics

A total of 223 patients were recruited in this study. The median age of all patients was 54.3 years (range 24-72 years). Of these patients, 129 received integrated IMRT and 94 patients received 3D-CRT segmented regimen as post-mastectomy radiation therapy. Regarding postoperative chemotherapy, 144 patients completed anthracycline-based regimen EC followed by taxanes and 79 patients completed anthracycline-based regimen FEC followed by taxanes. Patient characteristics and treatments are summarized in Table 1.

### Survival

**Figure 1:**
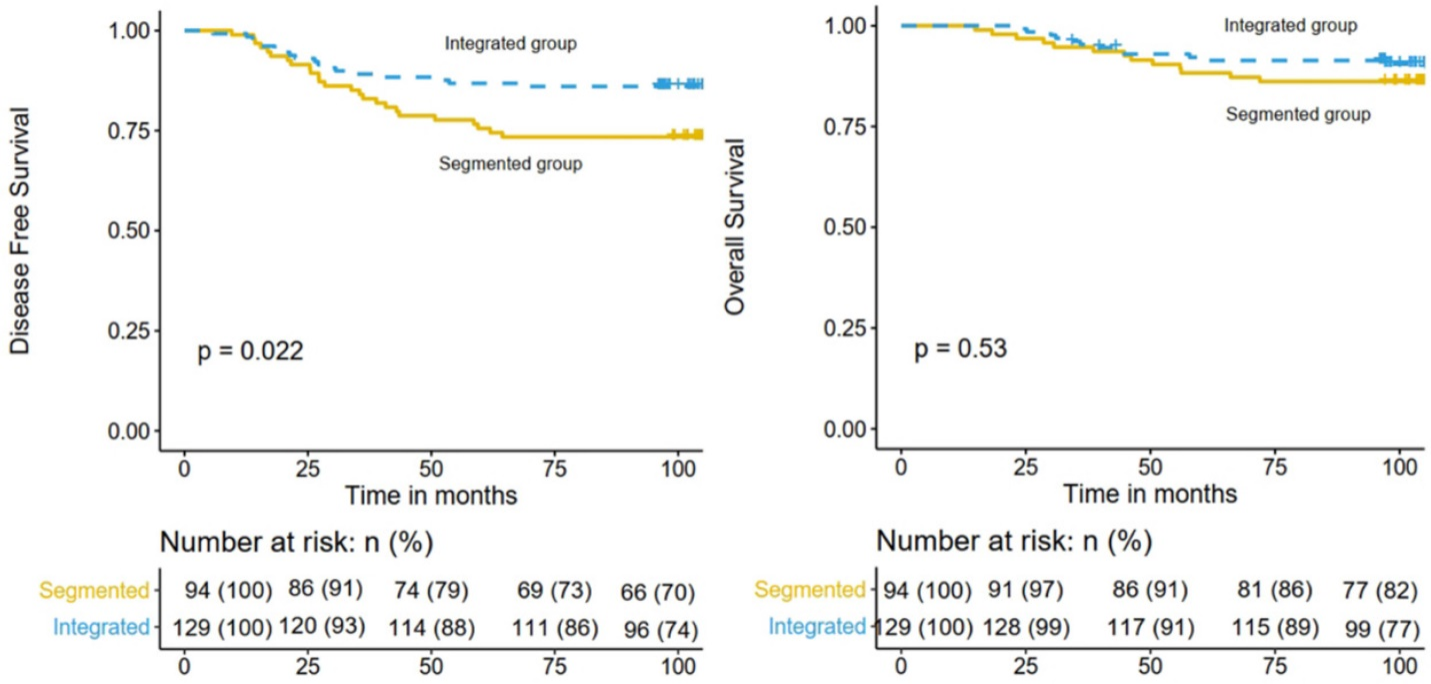
Comparison of DFS (left) and OS (right) in the integrated IMRT group and the 3D-CRT segmented regimen group.

The median follow-up time was 104.3 months (range 23.1-131.7 months). By the time of the last follow-up, recurrence of any type occurred in 43 patients and 27 patients died. For the whole cohort, the 5-year DFS and OS rates were 82.1% (95% confidence interval [CI], 77.2%-87.3%) and 90.1% (95% CI, 86.2%-94.1%), respectively. And the 8-year DFS and OS rates were 80.7% (95% CI, 75.7%-86.1%) and 89.2% (95% CI, 85.2%-93.4%), respectively. The 8-year DFS rates of the integrated IMRT and 3D-CRT segmented regimen groups were 86.0% (95% CI, 80.3%-92.2%) and 73.4% (95% CI, 65.0%-82.9%), respectively (*P* = 0.022; Figure 1 left), while the 8-year OS rates were 91.4% (95% CI, 86.6%-96.4%) and 86.2% (95% CI, 80.3%-92.2%), respectively (*P* = 0.530; Fig. 1 right).

Multivariate analysis demonstrated that radiotherapy was an independent prognostic factor for DFS (hazard ratio [HR], 0.53; 95% CI, 0.29-0.97; *P* = 0.041). In addition to the radiotherapy treatment, IMNI was independent prognostic factor for DFS (P < 0.05). Differentiation and IMNI were independently associated with decreased OS (P < 0.05). The results of the univariate and multivariate analyses for DFS and OS are summarized in Table 2.

### Patterns of recurrence

The patterns of any type of tumor recurrence are presented in Table 3. In total, 43 patients experienced tumor recurrence. Recurrent diseases in these patients were detected with radiologic imaging, and 20 patients confirmed with histopathology. Distant metastasis was the main type of recurrence; it occurred in 32 patients, including 10 patients with LRR. LRR rates were low in the two groups, with 2.3% for the integrated IMRT group and 7.4% for the 3D-CRT segmented regimen group.

### Chronic toxicity

Chronic skin toxicity, ipsilateral lung injury, cardiac injury, and ipsilateral shoulder mobility were observed. The numbers of patients that suffered from chronic skin toxicity including fibrosis, atrophy, retraction, telangiectasia, and hyperpigmentation in the integrated IMRT and 3D-CRT segmented regimen groups, were 96 and 73, respectively (*P* = 0.577). Most of these patients had grade 1-2 skin reaction. The percentages of patients who suffered from grade 1-2 ipsilateral lung injury in the integrated IMRT group and the 3D-CRT segmented regimen group were 30.2% and 31.9%, respectively (*P* = 0.788). The percentages of patients with left breast cancer who suffered from grade 1-2 cardiac injury in the integrated IMRT and the 3D-CRT segmented regimen groups were 30.6% (30/98) and 25.3% (19/75), respectively. No difference was observed in grade 1-2 ipsilateral shoulder mobility between the integrated IMRT and 3D-CRT segmented regimen groups (46.5% vs. 47.9%; *P* = 0.841). Chronic toxicities are summarized in Table 4.

### Heart and lung dosimetry

For the whole cohort, the mean heart dose for all patients was 3.6 Gy (range 0.5-6.2 Gy); it was 4.9 Gy (range 0.7-5.5 Gy) for patients with left-sided breast cancer and 0.8 Gy (range 0.6-2.1 Gy) for patients with right-sided breast cancer. Lung doses V20, V10, and V5 were 17.23%, 32.21%, and 51.54% respectively. No significant heart and lung dosimetry differences were observed between the two treatment groups.

## Discussion

A recent meta-analysis published by the Early Breast Cancer Trialists' Collaborative Group (EBCTCG) revealed that locoregional irradiation reduced the rates of recurrences and increased significantly the survival for breast cancer patients who had positive axillary nodes after modified radical mastectomy 6. Post-mastectomy radiation therapy plays an important role in the management of breast cancer. Although chest wall and regional nodes delineation techniques have been discussed with available contouring guidelines, treatments of chest wall and nodal regions contoured as a whole PTV in the planning computed tomography have not yet been accepted as a routine practice. 3D-CRT still serves as a common treatment in post-mastectomy radiation therapy. However, due to the use of mixed beams for regional nodes and the limitation of the depth of the electron beam, the conformity and uniformity of the CTVs may not be adequate. Moreover, the junction or overlap between the tangents and anterior fields causes an inhomogeneous dose distribution to the tissue between the chest wall and the supraclavicular region +/- IMN, which may reduce the tumor control and increase the normal tissues toxicities. Computed tomography-based treatment planning has been accepted widely and found to offer better coverage of supra/infraclavicular targets in a previous dosimetric study 7. Our previous study explored the dosimetric characteristics and acute adverse effects of irradiation of the chest wall and supraclavicular region contoured as an integrated volume with IMRT after modified radical mastectomy 5. We found that integrated IMRT for the target volume of the chest wall and supraclavicular region had dosimetric advantages and tolerable acute adverse effects compared to conventional segmented 3D-CRT. This long-term follow-up study was conducted to further evaluate the efficacy differences of the two radiotherapy techniques for post-mastectomy breast cancer patients.

Based on the results of this study, radiotherapy treatment was independently associated with a decreased recurrence rate after modified radical mastectomy. Irradiation regimen of the chest wall and supraclavicular region as an integrated volume with IMRT could improve DFS to 86.0% at 8 years, while conventional segmented 3D-CRT could improve DFS to 73.4% at 8 years (*P* = 0.022). However, improved DFS did not translate into a prolonged OS, with an 8-year OS of 91.4% for patients treated with integrated IMRT and 86.2% for patients treated with segmented 3D-CRT (*P* = 0.530). The effect of post-mastectomy radiation therapy on OS was not as significant as its effect on DFS. Several reasons may account for this phenomenon, such as the short follow-up time and the limited sample size. Meta-analysis of the EBCTCG including 8135 patients with a 20-year follow-up showed a statistically significant benefit of post-mastectomy radiation therapy for OS of breast cancer patients 6. The meta-analysis also demonstrated that the effect of post-mastectomy radiation therapy on mortality starts at 10 years out. Cardiovascular toxicity induced by left-sided radiotherapy or IMNI may compromise the survival benefit. A previous study reported that the cumulative incidence of a major coronary event increased by 7.4% per Gy within 20 years of follow-up 8. However, no severe cardiovascular toxicity was observed between the two groups.

Interestingly, IMNI was an independent prognostic factor for DFS (HR 3.45; 95% CI, 1.21-5.31; *P* = 0.013) and OS (HR 0.53; 95% CI, 1.03-5.79; *P* = 0.048) in our study. This is similar to a result reported from a recent retrospective research conducted by Luo et al. They evaluated the efficacy of IMNI in 497 breast cancer patients, stage II-III, after preoperative systemic therapy and surgery. They found that IMNI was an independent prognostic factor for DFS (*P* = 0.014) and OS (*P* = 0.047) in matched patients 9. Moreover, the DBCG-IMN, MA.20, and EORTC 22922/10925 trials suggested that post-operative patients could benefit from IMNI 10-12.

Toxicities induced by post-mastectomy radiation therapy are divided into acute (occurring within 3 months of radiotherapy) and chronic. No significant differences in acute side effects were observed between the two regimen groups in our previous study 5. Chronic skin toxicity including fibrosis, atrophy, hetraction, helangiectasia, and hyperpigmentation, ipsilateral lung injury, cardiac injury, and ipsilateral shoulder mobility were observed. No significant differences in these chronic side effects were observed between the two regimen groups. Among these chronic toxicities, the radiation induced cardiac injury has remained a concern because IMNI and/or left-sided radiotherapy increase the radiation dose to the heart 13. The mean heart dose for all patients was 3.6 Gy (range 0.5-6.2 Gy); it was 4.9 Gy (range 0.7-5.5 Gy) for patients with left-sided breast cancer and 0.8 Gy (range 0.6-2.1 Gy) for patients with right-sided breast cancer. IMNI and/or left-sided radiotherapy increase the radiation dose to the heart, and irradiation of the heart in left-sided breast RT has been showed to have association with an increased risk of cardiac events 12. However, recent large-scale studies with long term follow-up demonstrated that left-side radiation and IMNI did not increase cardiovascular mortality 10, 11, 14. Given the debate, patients treated with IMNI or/and left-sided radiotherapy was picked up when accessing cardiac injury to observe the relationship between the IMNI and survival or chronic heart toxicity in this study. Additionally, with the development of radiotherapy technologies, cardiotoxicity will decline over time 15. Therefore, continued follow-up of these studies may be needed to confirm the effect of cardiac toxicity on OS, due to the limited cardiovascular events.

In conclusion, this study is the first article to report the results of long term follow up survival among the post mastectomy breast cancer patients. Our study suggests that IMRT for treating chest wall and regional nodes contoured as a whole PTV could reduce the recurrence rate for post-mastectomy breast cancer patients with tolerable toxicities. Due to the retrospective nature of the study and the limited number of patients recruited, future prospective randomized trials are warranted to confirm the benefit of DFS and its long-term effects on survival and toxicity.

## Figures and Tables

**Table 1:** Patient, tumor and treatment characteristics

	Integrated group	Segmented group	*p*-Value
	n=129	n=94	
**Age**			0.418
Mean (years)	53.9	54.6	
Range	29-72	24-69	
**Menopausal status**			0.288
Premenopausal	69	57	
Postmenopausal	60	37	
**Laterality**			0.379
Right	57	36	
Left	72	58	
**Tumor location**			0.351
Medial/central	39	34	
Outer	90	60	
**Pathology**			0.607
Ductal carcinoma	124	89	
Lobular carcinoma	2	2	
Others	3	3	
**Estrogen receptor status**			0.621
Negative	24	20	
Positive	105	74	
**Progesterone receptor status**			0.207
Negative	23	25	
Positive	106	69	
**HER2 overexpression**			0.361
Negative	109	75	
Positive	20	19	
**Triple negative**			0.354
Yes	16	8	
No	113	86	
**Differentiation**			0.628
well	34	26	
moderate	67	45	
poor	28	23	
**Clinical T stage**			0.305
1-2	101	68	
3-4	28	26	
**Clinical N stage**			0.699
1	15	17	
2	80	56	
3	24	21	
**Lymph-vascular invasion**			0.753
Negative	59	41	
Positive	70	53	
**Internal mammary node irradiation**			0.675
Yes	53	36	
No	76	58	
**Hormone therapy**			0.342
Yes	103	70	
No	26	24	

**Table 2:** Univariate and multivariate analysis for disease-free survival and overall survival

Variables	UVA (DFS)			MVA (DFS)			UVA (OS)			MVA (OS)		
	HR	95% CI	*P*	HR	95% CI	*P*	HR	95% CI	*P*	HR	95% CI	*P*
Menopausal status (post- vs pre- )	0.91	(0.46-1.67)	0.763				1.19	(0.56-2.55)	0.636			
Laterality (left vs right)	0.83	(0.46-1.52)	0.551				0.97	(0.41-1.88)	0.737			
Tumor location (outer vs medial/central)	0.71	(0.39-1.32)	0.279				0.39	(0.24-1.09)	0.082	0.49	(0.23-1.05)	0.067
Clinical T stage (cT3-4 vs cT1-2)	1.77	(0.94-3.31)	0.076	1.38	(0.72-2.65)	0.331	1.67	(0.69-3.48)	0.273			
Clinical N stage (cN3 vs cN1-2)	2.06	(1.13-3.78)	0.019	1.81	(0.96-3.40)	0.066	2.41	(1.04-4.77)	0.038	2.03	(0.95-4.37)	0.068
Lymph-vascular invasion (positive vs negative)	1.28	(0.71-2.33)	0.415				0.77	(0.39-1.83)	0.675			
Estrogen receptor (positive vs negative)	0.59	(0.33-1.09)	0.095				0.58	(0.25-1.17)	0.117			
Progesterone receptor (positive vs negative)	0.89	(0.44-1.79)	0.745				1.19	(0.48-3.31)	0.647			
HER2 overexpression (positive vs negative)	1.11	(0.52-2.41)	0.786				0.59	(0.41-2.79)	0.909			
Differentiation (poor vs well/moderate)	1.75	(0.92-3.30)	0.087	1.44	(0.76-2.74)	0.268	1.54	(1.31-5.99)	0.007	2.53	(1.18-5.46)	0.017
Internal mammary node irradiation (no vs yes)	2.68	(1.29-5.60)	0.009	3.45	(1.21-5.31)	0.013	3.12	1.01-6.09)	0.049	2.33	(1.03-5.79)	0.048
Radiotherapy (integrated vs segmented)	0.51	(0.28-0.94)	0.029	0.53	(0.29-0.97)	0.041	0.81	(0.38-1.71)	0.566			

*Abbreviations:* CI: confidence interval; DFS: disease-free survival; HER2: human epidermal growth factor receptor 2; HR: hazard ratio; MVA: multivariate analysis; OS: overall survival; UVA: univariate analysis.

**Table 3:** Patterns of recurrence

	Total (%)	Integrated group (%)	Segmented group (%)	*P*
Patients	223 (100)	129 (100)	94 (100)	
Recurrence	43 (19.3)	18 (13.9)	25 (26.6)	0.018
Local	7 (3.1)	3 (2.3)	4 (7.4)	
Regional Axillary	5 (2.2)	2 (1.6)	3 (3.2)	
Supraclavicular	6 (2.7)	3 (2.3)	3 (3.2)	
Distant	32 (14.3)	13 (10.1)	19 (20.2)	

**Table 4:** Chronic toxicity outcomes

Toxicity	Integrated group (%)		Segmented group (%)		P (Grade 1-2)
	Grade 1-2	Grade 3-4	Grade 1-2	Grade 3-4	
Fibrosis	59 (45.7)	5 (3.9)	42 (44.7)	3 (2.3)	0.876
Atrophy/Retraction	61 (47.3)	3 (2.3)	40 (42.6)	2 (2.1)	0.483
Telangiectasia	21 (16.3)	1 (0.8)	14 (14.9)	2 (2.1)	0.779
Hyperpigmentation	40 (31.0)	1 (0.8)	24 (25.5)	0 (0.0)	0.372
Ipsilateral lung injury	39 (30.2)	0 (0.0)	30 (31.9)	0 (0.0)	0.788
Cardiac injury (IMNI or/and left-sided radiotherapy)	30 (30.6)	0	19 (25.3)	0	0.445
Ipsilateral shoulder mobility	60 (46.5)	1 (0.8)	45 (47.9)	0 (0.0)	0.841

*Abbreviations*: IMNI = internal mammary node irradiation.

## Abbreviations

DFS: disease-free survival
OS: overall survival
IMRT: intensity-modulated radiotherapy
PTV: planning target volume
3D-CRT: 3-dimensional conformal radiotherapy
IMNI: internal mammary node irradiation
HER2: human epidermal growth factor receptor 2
CTVc: target volume for the chest wall
CTVs: target volume for the supraclavicular region
PTVc: planning target volume for the chest wall
PTVs: planning target volume for the supraclavicular region
LRR: locoregional recurrence
